# Drain Current Model for Double Gate Tunnel-FETs with InAs/Si Heterojunction and Source-Pocket Architecture

**DOI:** 10.3390/nano9020181

**Published:** 2019-02-01

**Authors:** Hongliang Lu, Bin Lu, Yuming Zhang, Yimen Zhang, Zhijun Lv

**Affiliations:** Key Laboratory of Wide Band-Gap Semiconductor technology, School of Microelectronics, Xidian University, Xi’an 710071, China; zhangym@xidian.edu.cn (Y.Z.); ymzhang@xidian.edu.cn (Y.Z.); zhijjunlv@163.com (Z.L.)

**Keywords:** TFET, BTBT, InAs/Si, heterojunction, staggered-bandgap, source-pocket, 2D Poisson equations, parabolic approximation, Kane’s model, current model

## Abstract

The practical use of tunnel field-effect transistors is retarded by the low on-state current. In this paper, the energy-band engineering of InAs/Si heterojunction and novel device structure of source-pocket concept are combined in a single tunnel field-effect transistor to extensively boost the device performance. The proposed device shows improved tunnel on-state current and subthreshold swing. In addition, analytical potential model for the proposed device is developed and tunneling current is also calculated. Good agreement of the modeled results with numerical simulations verifies the validation of our model. With significantly reduced simulation time while acceptable accuracy, the model would be helpful for the further investigation of TFET-based circuit simulations.

## 1. Introduction

Owing to the band-to-band tunneling (BTBT) mechanism, Tunnel field-effect transistors (TFETs) allow further scaling of operation voltages, which makes them the most promising alternatives to the conventional metal oxide semiconductor field-effect transistors (MOSFETs) for low-power applications [1,2,3,4]. However, the All-silicon TFET suffers from unacceptably low on-state current, which is even lower than the demand reported by the International Technology Roadmap for Semiconductors (ITRS) [5,6], due to the indirect and large bandgap and thus its practical use is retarded.

To address this issue, various design improvements [7,8,9] and 2D materials [10,11] have been proposed and heterojunction TFET (HTFET) made of Ⅲ-Ⅴ/Si have been studied as promising solution. Among all the Ⅲ-Ⅴ/Si structures, InAs/Si HTFET has been proposed for the highest tunneling current for the p-TFET due to its much lower tunneling mass [12], staggered band lineup and the direct tunneling process [13]. Besides that, with an ultra-thin doping pocket inserted between the heavily doped source and the intrinsic channel region [14,15], the source-pocket TFET (SP-TFET) was proposed for more abrupt tunnel junction and steeper energy band bending, resulting in reduced tunneling distance and improved on-state tunneling current. The energy-band engineering of InAs/Si heterojunction and the novel device structure of SP concept improving the device performance have motivated us to combine both the techniques in a single device to further boost up the characteristics and the InAs/Si HSP-TFET is proposed in this paper. The proposed InAs/Si HSP-TFETs can significantly enhance the device performance.

On the other hand, TFET devices so far are mainly studied by the aid of TCAD simulator, an analytical model which is helpful to provide fast results for circuit simulations is still in ample necessity. Some models [16,17,18] are developed for conventional TFET structures. However, in order to simplify the integration process, the tunneling volume is assumed to be the device volume which is unchanged with the gate voltage. Although the electric field is very large near the source/channel interface, the energy band do not overlap in the region where the distance from the source/channel interface is less than the shortest tunneling distance. Namely, the tunneling window is not open. Thus no BTBT happens. Considering that the BTBT rate changes very rapidly, integrating the BTBT rate over the unchanged device volume sums extremely large but actually non-existent BTBT rate and the current would be overestimated. These models present a brief insight for the design of TFETs, but the simplification is not exactly suitable for the actual physical situation, which would lead to improper results sometimes. 

In this paper, we proposed the InAs/Si HSP-TFETs combing InAs/Si heterojunction and the SP technique to improve device performance and study their impact in a single device. Furthermore, an analytical potential model for the proposed device is developed. Based on the potential model, the shortest tunnel distance considering the variation of the tunneling volume with the gate voltage is also presented and the current is calculated by numerically integrating the carrier tunneling rate over the varied tunnel volume. The paper is arranged as follows: Section 2 exhibits the mechanism of the proposed InAs/Si HSP-TFET. The model derivation of this work is shown in Section 3. The results and discussions are shown in Sect 4 and Sect 5 gives the conclusion of this work.

## 2. Heterojunction Source-Pocket DGTFET

**Figure 1:**
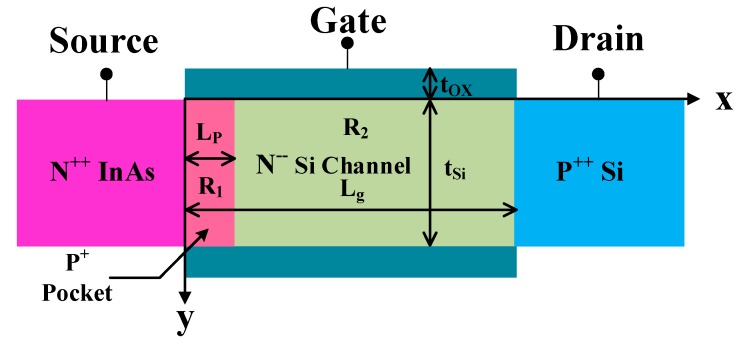
Schematic cross-sectional view of a p-type HSP-TFET.

The cross section view of the studied InAs/Si HSP-TFET and the coordinate system adopted in this work are shown in Figure 1. The tunneling junction between the N++ source region and the narrow P+ pocket acts as a carrier source for the channel. The device channel can include two regions, as shown in Figure 1, which are the source pocket region denoted as R_1_ and the rest of channel denoted as R_2_. Typical values of the device and material parameters used are listed in Table A1 and Table A2 if not otherwise stated. 

**Figure 2:**
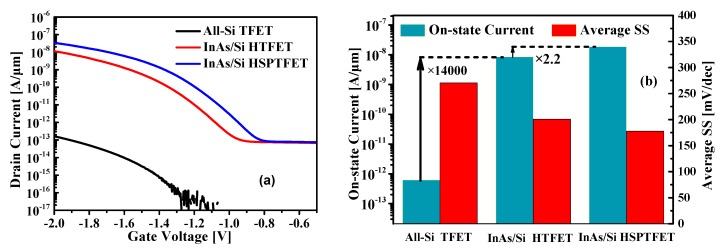
(**a**) Simulated I_D_-V_G_ curves for different TFET configurations. (**b**) Plots for comparison of the on-state current and SS. The HSP-TFET is with the pocket doping N_P_ = 1 × 10^19^ cm^−3^ and the pocket length is L_P_ = 6 nm. The gate work function is 4.3 eV and drain bias is −0.5 V.

The effect of the staggered bandgap of InAs/Si heterojunction and the thin fully depleted pocket reduce the tunneling distance and result in more efficient carrier injection from the source region to the channel body. Figure 2 shows the comparison in terms of transfer characteristics, on-state current and average subthreshold swing (SS). The on-state current is extracted at |V_GS_-V_tunnel_| = 1 V, where V_GS_ is the gate-to-source voltage, and V_tunnel_ is the gate voltage at which the drain current starts to be higher than the reverse leakage current [19]. The average SS is evaluated between V_tunnel_ and |V_GS_- V_tunnel_| = 1 V. It is shown that the All-Si TFET has an extremely low off-state current (<0.5 fA/μm), but its on-state current is less than 0.5 pA/μm at V_GS_ of 2 V. By applying the staggered bandgap of InAs/Si heterojunction, extraordinary device improvement about a 14000-fold can be obtained in the on-state current with much smaller average SS for the InAs/Si HTFET, compared with the All-Si TFET counterpart. Moreover, by the aid of the heavily doped pocket, the drain current of the HSP-TFET can be further improved by 2.2 times higher than that of the HTFET without SP. It should be pointed out that despite of HSP-TFET simultaneously increased off-state current, it is still much smaller than that of a state-of-the-art 65-nm CMOS transistor which is about 10^−11^ A/μm [20].

**Figure 3:**
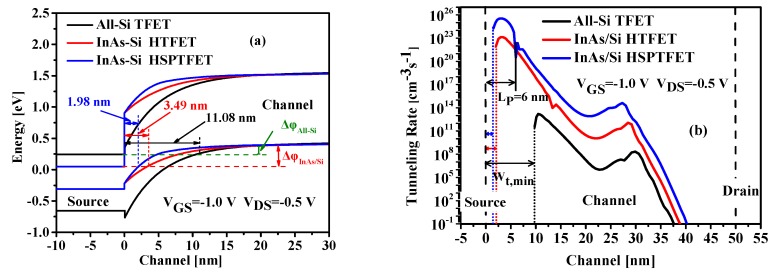
(**a**) Illustration of the energy band diagrams and (**b**) carrier band to band tunneling rate profile along the channel/oxide interface.

Figure 3 shows the simulated band diagram of InAs/Si HSP-TFET compared with InAs/Si HTFET and All-Si TFET counterparts. The energy band of All-Si TFET has been moved up-forward for the band alignment in drain region with that of InAs/Si HSP-TFET. The large negative gate voltage drop results in an overlap between the channel valence band and the source conductance band. Thus, carriers in the interval of channel valence band edge and source conductance band edge, namely the tunnel window Δφ, can be tunneled from the source conductance band to the channel valence band. As only the carriers in Δφ can tunnel from the source into the channel, the carrier energy distribution is limited and the high energy carriers are effectively filtered. Thus the electronic system is much more immune to the hot carriers compared with a conventional MOSFET [18].

The shortest tunnel distance W_t,min_ between available source conduction band states and channel valence band states must appear nearby the highest electric field and is one of the most significant parameters in Figure 3a. W_t,min_ determines the lower bound of the tunnel volume. The more smaller W_t,min_, the larger the tunnel volume. Owing to the smaller bandgap of InAs and the large conduction band offset at InAs/Si interface, InAs/Si HTFETs shows much smaller W_t,min_ (3.49 nm) compared with that (11.08 nm) of All-Si TFETs. Thus much higher tunneling rate and larger tunnel volume are obtained for InAs/Si HTFETs, as shown in Figure 3b, which is consistent with the extraordinary improvement of the on-state current. The W_t,min_ of InAs/Si HTFET can be further reduced from 3.49 nm to 1.98 nm by applying the heavily doped SP structure as plotted in Figure 3a. Figure 3b shows that the InAs/Si HSP-TFET has greatly elevated tunneling rate, especially over the pocket region, compared with InAs/Si HTFETs and this is the reason of the highest tunnel on-state current for the InAs/Si HSP-TFET as shown in Figure 2.

## 3. Model Derivation for InAs/Si HSP-TFET

### 3.1. Channel Potential Model

With the assumption of a fully depleted channel in the subthreshold operation domain, the charge density in the channel region is equal to the ionized doping concentration. Thus the 2D Poisson equations can be used to describe the potential distribution across the regions R_1_ and R_2_ as Equation (1) [21].

(1) $$\frac{\partial^{2}\Phi_{j}\left(x,y\right)}{\partial x^{2}}+\frac{\partial^{2}\Phi_{j}\left(x,y\right)}{\partial y^{2}}=\frac{qN_{i,j}}{\varepsilon_{Si}},$$

where Φ_j_(x,y) is the overall channel potential and N_i,j_ is the doping concentration. The subscript j = 1, 2 for regions R_1_ and R_2_, respectively. 

The parabolic approximation [22,23] of the potential in the direction normal to the channel surface is adopted, so that the channel potential can be described as an analytical Equation (2).

(2) $$\Phi_{j}\left(x,y\right)=\varphi_{j}\left(x\right)+C_{j,1}\left(x\right)y+C_{j,2}\left(x\right)y^{2},$$

Here the C_1,j_(x) and C_2,j_(x) are arbitrary functions of the surface potential φ_j_(x). At the interface between the channel and the oxide, the electric flux must be continuous. In addition, the electric field in the middle position of the channel along the y-direction equals to zero due to the structure symmetry of device.

(3) $$\left\{ \begin{array}{l} \frac{d\Phi_{j}\left(x,y\right)}{dy}|{}_{y=0}=-\frac{\varepsilon_{OX}}{\varepsilon_{Si}}\left(\frac{V_{gs,j}-\varphi_{j}\left(x\right)}{t_{OX}}\right),\\ \frac{d\Phi_{j}\left(x,y\right)}{dy}|{}_{y=\frac{t_{Si}}{2}}=0, \end{array}\right.$$

where V_gs,j_ = V_GS_ − V_FB,j_. V_FB,j_ is the gate flat band voltage. With Equation (3), the coefficient C_1,j_(x) and C_2,j_(x) can be expressed as functions of the surface potential φ_j_(x).

(4) $$\left\{ \begin{array}{l} C_{1,j}\left(x\right)=-\frac{\varepsilon_{OX}}{\varepsilon_{Si}t_{OX}}\left(V_{gs,j}-\varphi_{j}\left(x\right)\right),\\ C_{2,j}\left(x\right)=+\frac{\varepsilon_{OX}}{\varepsilon_{Si}t_{OX}t_{Si}}\left(V_{gs,j}-\varphi_{j}\left(x\right)\right), \end{array}\right.$$

By substituting Equation (4), and Equation (2) into Equation (1), the 2D Poisson equations can be reduced to the well-known form.

(5) $$\frac{\partial^{2}\varphi_{j}\left(x\right)}{\partial x^{2}}-\beta^{2}\varphi_{j}\left(x\right)=\alpha_{j},$$

where β = (2ε_ox_/t_ox_t_si_ε_si_)^1/2^, α_j_ = qN_i,j_/ε_si_ − β^2^V_gs,j_.

The general solutions for Equation (5) can be expressed as Equation (6).

(6) $$\varphi_{j}\left(x\right)=A_{j}\exp\left(+\beta x\right)+B_{j}\exp\left(-\beta x\right)-\frac{\alpha_{j}}{\beta^{2}},$$

Undetermined parameters A_j_ and B_j_ in Equation (6) can be solved by the boundary conditions, i.e., the potential and electric displacement continuities at the Source/R_1_, R_1_/R_2_ and R_2_/Drain interfaces.

(7) {φ1(0)=VS+VtIn(NSni,Source)+ΔECq+ΔVRef,φ1(LP)=φ2(LP),∂φ1(x)∂x|x=LP=∂φ2(x)∂x|x=LP,φ2(Lg)=VD−VtIn(NDni,Drain),

where ΔV_Ref_ = (E_g,Channel_ − E_g,Source_)/2q and ΔE_C_ = χ_Channel_ − χ_Source_ is the conductance band offset of the InAs/Si heterojunction interface. n_i,Source_ and n_i,Drain_ are the intrinsic carrier density of source and drain regions, respectively. V_t_ is the thermal voltage. V_S_ and V_D_ are source and drain bias respectively. The A_j_ and B_j_ are solved by substituting Equation (7) into Equation (6), and the results are given in Equation (8).

(8) $$\left\{ \begin{array}{rr} A_{1}= & \left(1-M\right)\times \gamma_{1}+N\times \gamma_{2}-C_{A1}\times \gamma_{3},\\ B_{1}= & M\times \gamma_{1}-N\times \gamma_{2}+C_{B1}\times \gamma_{3},\\ A_{2}= & \left(1-M\right)\times \gamma_{1}+N\times \gamma_{2}-C_{A2}\times \gamma_{3},\\ B_{2}= & M\times \gamma_{1}-N\times \gamma_{2}+C_{B2}\times \gamma_{3}, \end{array}\right.$$

With

(9) $$\left\{ \begin{array}{l} C_{A1}=\left(\theta_{1}^{2}+\theta_{2}^{2}\right)/FM,\\ C_{B1}=C_{A1},\\ C_{A2}=\left(1+\theta_{2}^{2}\right)/FM,\\ C_{B2}=\left(1+\theta_{2}^{2}\right)\theta_{1}^{2}/FM,\\ FM=2\theta_{2}\left(1-\theta_{1}^{2}\right), \end{array}\right.\left\{ \begin{array}{l} \gamma_{1}=\frac{\alpha_{1}}{\beta^{2}}+\Phi_{1}\left(0,0\right),\\ \gamma_{2}=\frac{\alpha_{2}}{\beta^{2}}+\Phi_{2}\left(L_{g},0\right),\\ \gamma_{3}=\frac{\alpha_{1}-\alpha_{2}}{\beta^{2}}, \end{array}\right.\left\{ \begin{array}{l} M=\frac{\theta_{1}^{2}}{\theta_{1}^{2}-1},\\ N=\frac{\theta_{1}^{}}{\theta_{1}^{2}-1}, \end{array}\right.$$

where θ_1_ = exp(β×L_g_) and θ_2_ = exp(β×L_P_). Φ_1_(0,0) and Φ_2_(L_g_,0) are surface potential at x = 0 and x = L_g_ respectively. 

With the obtained surface potential φ_j_(x), the overall channel potential Φ_j_(x,y) can be obtained by Equation (2). Differentiating the channel potential, the electric-field distribution in the channel region can be obtained.

(10) $$\left\{ \begin{array}{l} E(x,y)=\sqrt{E_{jx}^{2}+E_{jy}^{2}},\\ E_{jx}=\frac{\partial \Phi_{j}\left(x,y\right)}{\partial x}=\left[-\frac{\beta^{2}}{2}y^{2}+\frac{\beta^{2}t_{Si}}{2}y+1\right]\frac{\partial \varphi_{j}\left(x\right)}{\partial x},\\ E_{jy}=\frac{\partial \Phi_{j}\left(x,y\right)}{\partial y}=\beta^{2}\left[y-\frac{t_{Si}}{2}\right]\left[V_{gs,j}-\varphi_{j}(x)\right], \end{array}\right.$$

where E(x,y) is the magnitude of the electric field. E_x_ and E_y_ are electric field along the x and y directions, respectively. The energy bands are derived from the potential expression Equation (11).

(11) $$\left\{ \begin{array}{ll} E_{C,Source} & =\left(-q\right)\times \Phi_{1}\left(0,0\right),\\ E_{C,j} & =\left(-q\right)\times \Phi_{j}\left(x,y\right)\;+\left(\chi_{S\mathrm{ource}}-\chi_{Channel}\right),\\ E_{C,Drain} & =\left(-q\right)\times \Phi_{2}\left(L_{g},0\right)+\left(\chi_{S\mathrm{ource}}-\chi_{Drain}\right),\\ E_{V,Source} & =E_{C,Source}-E_{g,Source},\\ E_{V,j} & =E_{C,j}-E_{g,Channel},\\ E_{V,Drain} & =E_{C,Drain}-E_{g,Drain}, \end{array}\right.$$

### 3.2. Drain Current Model

The W_t,min_ can be used to determine the tunnel volume and calculate the device tunneling current. The carrier tunnel into the channel as the source conduction band and the channel valence band are in-line to each other. Hence, the W_t,min_ can be obtained as E_C,Source_ = E_V,j_(W_t,min_,y).

(12) $$W_{t,\min}=\frac{1}{\beta }\ln\left(Cons+\alpha_{j}/\beta^{2}-\sqrt{{\left(Cons+\alpha_{j}/\beta^{2}\right)}^{2}-4A_{j}B_{j}}/2A_{j}\right),$$

With

(13) $$Cons=\frac{\left(\Phi_{1}\left(0,0\right)+\chi_{Source}-\chi_{Channel}-E_{g,Channel}\right)-0.5\beta^{2}\left(y^{2}-t_{Si}y\right)V_{gs,j}}{1-0.5\beta^{2}\left(y^{2}-t_{Si}y\right)},$$

The W_t,min_ reduction caused by the increased gate bias boosts the tunneling current due to the larger tunneling volume. Instead of a full quantum treatment, the tunneling carriers are modeled by a generation-recombination process. For a given tunneling path of length L which starts at x = 0 and ends at x = L, holes are generated at x = 0 and electrons are generated at x = L [24]. Thus, the carrier tunneling rate can be equivalently processed by the generation rate and the tunnel current density of carriers tunneling from the source to the channel equals to electron charge times the integral of the generation rate G_BTBT_. Then the tunnel current can be computed by integrating the tunnel current density over tunnel volume.

Considering the exponential decrease of the tunneling rate with the tunnel distance [25], the BTBT process from source to channel is assumed to be extended up to the channel center and the BTBT process from channel to drain usually known as the ambipolar effect [26] is limited in the right part of the channel. Thus the tunnel current can be described by Equation (14).

(14) {IBTBT,S−C=q∫0tSi(∫Wt,minLg2GBTBT(x,y)dx)dy,IBTBT,C−D=q∫0tSi(∫Lg2LgGBTBT(x,y)dx)dy,IBTBT=IBTBT,S−C+IBTBT,C−D,

The well-known Kane’s Model [27] is used to calculate the generation rate as Equation (15).

(15) $$G_{BTBT}\left(x,y\right)=A_{K}{\left|\frac{E}{E_{0}}\right|}^{2}\exp\left(-\frac{B_{K}}{\left|E\right|}\right),$$

In Equation (15), E_0_ = 1 V/cm and |E| is the electric field magnitude given by Equation (10). The A_K_ and B_K_ are the Kane’s tunneling parameters.

## 4. Results and Discussion

In this part, the numerical simulations by ISE TCAD tool from Synopsys were carried out to verify the validity of our model. In this work, a non-local path BTBT model along with SRH recombination and bandgap narrowing has been employed for carrier transport.

### 4.1. Channel Potential

**Figure 4:**
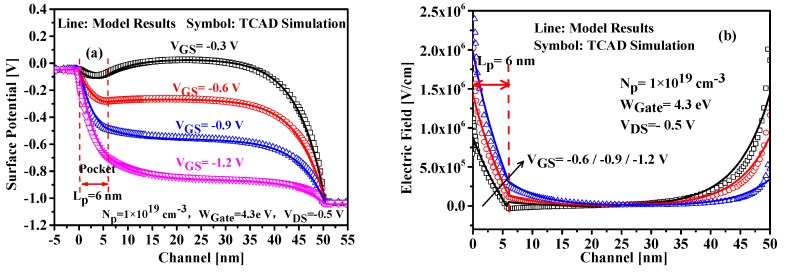
Variation of (**a**) the surface potential and (**b**) the lateral electric field with V_GS_.

Calculating with the proposed model, the channel surface potential and electric field versus gate voltage along the x-direction are plotted in Figure 4, in which the comparison with the results simulated by TCAD tool are also shown in this figure, and the excellent agreement demonstrates the validation of our model. Owing to the non-uniform modulation of the gate voltage on the channel potential, the potential near the tunneling junction becomes steeper with the increased gate voltage, leading to much larger electric field, which can be seen in Figure 4b. Furthermore, the potential change with the increased gate voltage greatly narrows the tunneling distance for the charge carriers, thus resulting in elevating tunnel probability.

**Figure 5:**
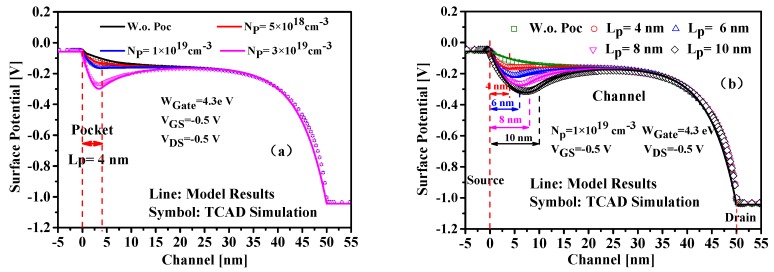
Surface Potential along the channel with (**a**) the pocket doping concentrations and (**b**) the pocket lengths.

The surface potential distributions of InAs/Si HSP-TFET with different SP doping concentrations are plotted in Figure 5a. Increasing the SP doping concentration results in much more steeper surface potential distribution close to the source end and thus leads to improved electric field and higher drain tunnel current. But the carrier would be injected by diffusion over the barrier with a too heavily doped pocket structure. Thus, the SP doping concentration is fixed at 1 × 10^19^ cm^−3^ to guarantee that the carrier transport based on BTBT mechanism. Another critical parameter for the device design is the pocket length and Figure 5b shows the variation of the surface potential with pocket lengths. It can be seen that longer length is helpful to increase the steepness of the potential profile near the source end and therefore will lead to improved on-state tunnel current. The pocket length should be carefully designed because the pocket region with a fixed pocket doping density no longer remains fully depleted if it is too long and in this case, the subthreshold characteristics would be drastically degraded.

Excellent agreement of modeled potential results with the TCAD simulation exhibited in Figure 5 reveals that the presented potential model can predict the impact of pocket doping concentration and pocket length on the potential distribution with good accuracy. It should be noted that although the model is derived for the proposed InAs/Si HSP-TFET, it can also be extended to the homojunction DG-TFET, with correct parameters in Table A2.

### 4.2. Drain Current

**Figure 6:**
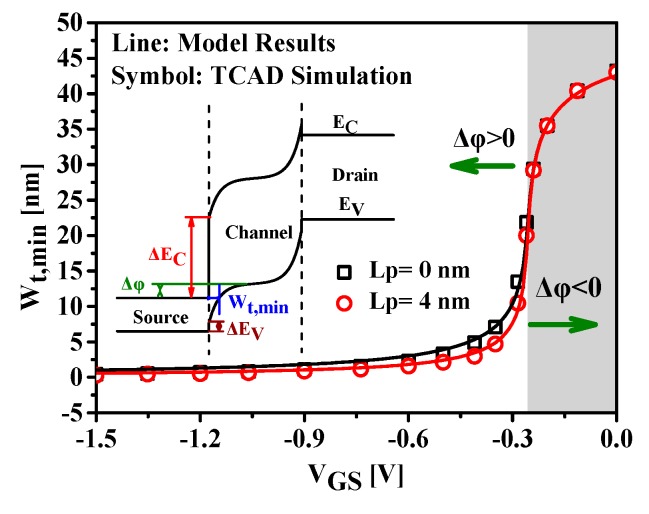
Shortest tunneling distance versus gate voltage. The pocket doping density for HSP-TFET is 1 × 10^19^ cm^−3^. The gate work function is 4.7 eV and the drain bias is 0.5 V.

The variation of the shortest tunnel distance with the gate voltage is illustrated in Figure 6. The TCAD simulation results have been extracted by measuring the alignment points between the source conductance band and the channel valence band, and are very sensitive to the meshing strategy. In our simulation, a very fine mesh grid across the tunneling junction is used. The good agreement obtained between the modeled results and the numerical simulations validates the proposed model. It is evident that the tunnel path is reduced significantly as the gate voltage increases. Due to larger tunneling volume and improved electric field, the reduction of W_t,min_ with gate voltage improves tunneling current. 

The pocket length equal to zero in Figure 6 corresponds to the case of HTFET without SP structure. The HSP-TFET with SP structure shows smaller shortest tunneling distance over a large gate-to-source voltage range in the positive tunneling widow, compared with that of HTFET and leads to higher tunnel on-state current. However, in the off-state, corresponding to the region of negative tunneling window indicated by shading, the HSP-TFET exhibits the same tunneling distance with that of HTFET, which predicts almost the same off-state tunnel current. 

**Figure 7:**
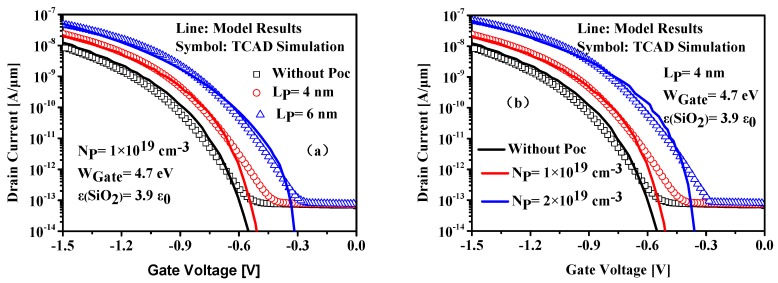
Transfer characteristics versus different (**a**) pocket doping concentrations and (**b**) pocket lengths.

Figure 7 shows the transfer characteristics calculated from our model for different pocket doping concentrations and pocket lengths. It is obvious that good agreement is obtained between the model results and the numerical simulations in the on-state where the BTBT current dominates. The large deviation in the off-state results from the neglect of the SRH thermal generation which dominates the off-state leakage current. Due to the heavily doped pocket structure, the InAs/Si HSP-TFET exhibits improved drain tunneling current compared with that of InAs/Si HTFETs without SP. This can be understood from Figure 5 in which the HSP-TFET shows a potential minimum near the source-to-channel tunneling junction caused by the inserted SP structure compared with HTFET without SP structure. The reduction of W_t,min_ and the increase of electric field due to the sharper potential profile help to boost the on-state tunnel current. 

**Figure 8:**
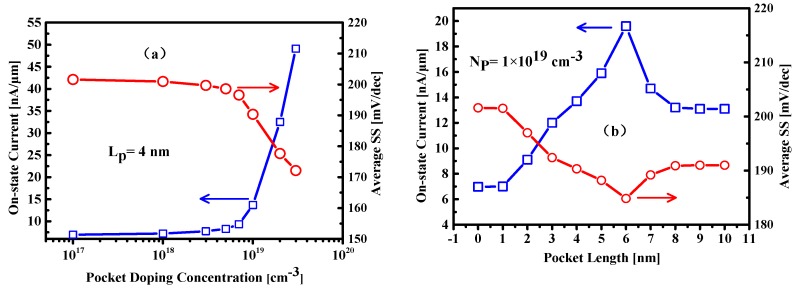
Plot of simulated on-state current and average SS as functions of (**a**) pocket doping concentration and (**b**) pocket length. The N_P_ = 1 × 10^17^ cm^−3^ and L_P_ = 0 nm correspond to the case of HTFET without SP structure.

The extracted on-state current and average SS, as functions of the source pocket doping and pocket length, are illustrated in Figure 8. Increasing the pocket doping concentration would cause the energy band to change more abruptly near the tunneling junction, as shown in Figure 5. and thus leads to elevated tunneling current and improved average SS. However, different behaviors of the on-state current and average SS versus the pocket length is exhibited in Figure 8b. It shows an optimum pocket length about 6 nm where the on-state current reaches maximum and the average SS reduces to minimum. For a fixed pocket doping, if the pocket length is increased and larger than the optimum value, the pocket no longer remains fully depleted and the mobile carriers in the pocket region screen the gate electric field, which would degrade the on-state current and also the subthreshold swing.

Finally, it is should be noted that although an ideally abrupt and defect/trap-free heterojunction between InAs and Si is assumed in this work to focus on a clear presentation of current model, they are important for the analysis of TFETs. The electrons can be excited from the valence band to the trap states arising from the very high lattice mismatch of about 11.6% between InAs and Si, and subsequent be emitted to the conduction band or vice versa, which leads to increased leakage current of TFETs [13,28]. In addition, this situation can be further degraded by the phonon-assisted tunneling via the trap sates in the bandgap and enhanced recombination process resulted from the traps at or close to the InAs/Si heterojunction [29]. Fortunately, these effects should not significantly affect our discussion, thus, the exclusion is reasonable in the initial stage of the model development. It also should be pointed out that this model is structure-dependent. It can be applied for TFETs made of double gate architecture even inclding other channel materials like germanium, as long as with correct parameters in Table A2.

## 5. Conclusions

In conclusion, a novel InAs/Si HSP-TFET is proposed to greatly enhance the device performance. The TCAD simulations reveal that the proposed structure shows an on-state current 28000 times higher than that of All-Si TFET and 2.2 times higher than that of InAs/Si HTFET with simultaneously improved subthreshold swing. An analytical potential model with numerically calculated drain current for the InAs/Si HSP-TFET is also developed. The proposed model provides very faster results with acceptable accuracy compared with TCAD simulations and would be helpful to the investigation of TFETs.

## Appendix A

**Table A1:** Device parameters used in the calculation and simulation.

Quantity	Value
Channel length, L_g_ (nm)	50
Pocket length, L_P_ (nm)	6
Source/Drain length, L_SD_ (nm)	50
Silicon layer thickness, t_Si_ (nm)	10
Gate dielectric thickness, t_ox_ (nm)	2
Source doping, N_s_ (cm^−3^)	1 × 10^20^
Pocket doping, N_P_ (cm^−3^)	1 × 10^19^
Channel doping, N_i_ (cm^−3^)	1 × 10^17^
Drain doping, N_D_ (cm^−3^)	1 × 10^21^

**Table A2:** Material parameters used in this work.

Quantity	Value
Vacuum permittivity	ε_0_
Silicon permittivity, ε_Si_	11.9ε_0_
Oxide permittivity, ε_OX_	3.9ε_0_
Source Energy band gap (InAs), E_g,Source_ (eV)	0.36
Channel Energy band gap (Si), E_g,Channel_ (eV)	1.12
Drain Energy band gap (Si), E_g,Drain_ (eV)	0.89
Source Affinity, χ_Source_ (eV)	4.93
Channel Affinity, χ_Channel_ (eV)	4.07
Drain Affinity, χ_Drain_ (eV)	4.18
Silicon Conduction density of states, N_C,Si_ (cm^−3^)	2.58 × 10^19^
Silicon Valance density of states, N_V,Si_ (cm^−3^)	1.94 × 10^19^
InAs Conduction density of states, N_C,InAs_ (cm^−3^)	8.72 × 10^16^
InAs Valance density of states, N_V,InAs_ (cm^−3^)	6.66 × 10^18^
